# Validation of the physical and RBE-weighted dose estimator based on PHITS coupled with a microdosimetric kinetic model for proton therapy

**DOI:** 10.1093/jrr/rrx057

**Published:** 2017-10-26

**Authors:** Kenta Takada, Tatsuhiko Sato, Hiroaki Kumada, Junichi Koketsu, Hideyuki Takei, Hideyuki Sakurai, Takeji Sakae

**Affiliations:** 1 Faculty of Medicine, University of Tsukuba, 1-1-1, Tennodai, Tsukuba, Ibaraki, 305-8575, Japan; 2 Japan Atomic Energy Agency, 2-4, Shirakata, Tokai, Ibaraki 319-1195, Japan; 3 Proton Beam Therapy Center, University of Tsukuba Hospital, 2-1-1, Amakubo, Tsukuba, Ibaraki, 305-8576, Japan

**Keywords:** proton therapy, relative biological effectiveness, PHITS, microdosimetric kinetic model

## Abstract

The microdosimetric kinetic model (MKM) is widely used for estimating relative biological effectiveness (RBE)-weighted doses for various radiotherapies because it can determine the surviving fraction of irradiated cells based on only the lineal energy distribution, and it is independent of the radiation type and ion species. However, the applicability of the method to proton therapy has not yet been investigated thoroughly. In this study, we validated the RBE-weighted dose calculated by the MKM in tandem with the Monte Carlo code PHITS for proton therapy by considering the complete simulation geometry of the clinical proton beam line. The physical dose, lineal energy distribution, and RBE-weighted dose for a 155 MeV mono-energetic and spread-out Bragg peak (SOBP) beam of 60 mm width were evaluated. In estimating the physical dose, the calculated depth dose distribution by irradiating the mono-energetic beam using PHITS was consistent with the data measured by a diode detector. A maximum difference of 3.1% in the depth distribution was observed for the SOBP beam. In the RBE-weighted dose validation, the calculated lineal energy distributions generally agreed well with the published measurement data. The calculated and measured RBE-weighted doses were in excellent agreement, except at the Bragg peak region of the mono-energetic beam, where the calculation overestimated the measured data by ~15%. This research has provided a computational microdosimetric approach based on a combination of PHITS and MKM for typical clinical proton beams. The developed RBE-estimator function has potential application in the treatment planning system for various radiotherapies.

## INTRODUCTION

Radiotherapy is used worldwide for non-invasive cancer treatment. Before performing radiotherapy, it is necessary to simulate the dose distribution in the patient’s body, known as treatment planning, through by a treatment planning system (TPS). In a TPS, the dose distribution in the target and normal tissue surrounding the target is estimated by a dose calculation algorithm. Although the dose calculation algorithm differs for each radiation type, Monte Carlo calculations are considered to be the most accurate. Monte Carlo simulations are employed in TPSs for radiotherapies such as X-ray therapy, charged particle therapy and boron neutron capture therapy (BNCT) [1–8].

Two different doses must be evaluated for charged particle therapy and BNCT: one is the physical dose, known as the absorbed dose, and the other is the biological dose, which takes into account the biological effectiveness of the radiation type. The biological dose is determined by multiplying the relative biological effectiveness (RBE) by the physical dose; thus, it is called the RBE-weighted dose. The RBE values employed in TPSs for carbon ion therapy were determined using the surviving fraction of cells as the biological end point. Several biophysical models for estimating the surviving fraction of cells irradiated by various radiation types have been developed, such as the local effect model (LEM) [9, 10], the repair-misrepair-fixation model [11], and the microdosimetric kinetic model (MKM) [12, 13].

The MKM is one of the most widely used models in radiation biology because it can determine the surviving fractions of cells irradiated by any type of radiation, based on the dose distribution of the lineal energy [14], *y*, and it is independent of the radiation type and ion species. Thus, it has been used to estimate the RBE in various radiotherapies, such as proton therapy [15], carbon ion therapy [16–21], BNCT [22], and X-ray therapy [23]. In these studies, the dose distributions of *y* were evaluated by measurements based on the tissue-equivalent proportional counter (TEPC) or simulations based on Monte Carlo particle transport codes. Considering that it is impractical to measure the dose distribution of *y* for all irradiation conditions in realistic radiotherapy fields by TEPC, the use of MKM alongside Monte Carlo simulations is more suitable for TPS implementation. However, for proton beam therapy, only a few studies on simulation-based RBE estimation have been reported [8, 24]. One reason for this is that most clinical proton therapy facilities use a constant value of 1.1 as the clinical RBE. On the other hand, recent studies have reported some variation in the RBE value according to the depth of the clinical proton beam (i.e. the depth of the spread-out Bragg peak: SOBP) [25–29]. Therefore, multilateral approaches such as MKM coupled with various Monte Carlo simulations are required for precise estimation of RBE.

In this research, the Particle and Heavy Ion Transport code System (PHITS) [30] was coupled with MKM because it has a function to calculate the dose distribution of *y* in a short computational time, called the microdosimetric function [31, 32]. The accuracy of PHITS coupled with MKM for estimating the RBE-weighted dose has been examined for carbon ion therapy [17, 19] and BNCT [22], but not for proton therapy. For the validation, a full simulation reproducing the beam line of the Proton Medical Research Center (PMRC) at the University of Tsukuba [33] was performed. The physical doses as well as their *y* distributions along with beam penetration were calculated, and these were converted to the RBE-weighted dose using MKM. These simulation results were compared with the corresponding published experimental data [15].

Based on the results obtained in this study, the RBE-weighted dose in the clinical proton beam line can be estimated using PHITS coupled with MKM. This is expected to be a very useful tool for treatment planning in various clinical conditions.

## MATERIALS AND METHODS

This study first validated the physical dose estimated by PHITS, and then focused on the RBE-weighted dose estimated by PHITS coupled with MKM.

### Validation of physical dose with full mock-up simulation geometry of the clinical proton beam line

**Fig. 1.**
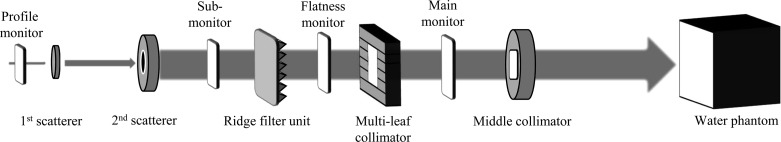
Calculation geometry used for the physical and RBE-weighted dose validation of the clinical proton beam therapy.

In the PHITS simulation, equipment such as a profile monitor, 1st scatterer, 2nd scatterer, sub-monitor, ridge filter, flatness monitor, multi-leaf collimator, main monitor, and middle collimator were placed upstream of the beam. All monitors were made of polyimide thin film with copper. The proton pencil beam was first broadened by the 1st scatterer, which was constructed from tungsten. The 2nd scatterer was made of lead alloy and plastic resin (acrylonitrile butadiene styrene). The ridge filter unit was made of aluminum alloy and was only used for the SOBP beam. In the simulation, each ridge-shaped bar was stacked as a multilayer structure, with thinner layers than the actual dimensions to calculate the influence of multiple Coulomb scattering in aluminum alloy more accurately. All collimators were made of brass. Patient-specific equipment (such as a range shifter, range compensator or patient collimator) was not considered (Fig 1).

The physical depth dose distribution produced by a 155 MeV beam was calculated by PHITS using the [T-deposit] function [34], which computes the deposition energy only from charged particles, i.e. the Kerma approximation was not employed in this study. The use of event generator mode is indispensable in the function. The nuclear reactions induced by neutrons above 20 MeV and protons were simulated by the intranuclear cascade model INCL4.6 [35], while those induced by neutrons below 20 MeV were simulated by the event generator mode version 2 [36] coupled with the nuclear data library JENDL-4.0 [37]. The angle straggling and energy straggling of protons are also considered by PHITS. The ATIMA model [38] was used for stopping power calculation. The EGS5 algorithm [39] was used for analyzing the motion of electrons, positrons and photons. The calculation mesh for the depth dose distribution had a step size of 2.5 mm in radius and 1 mm in depth. The simulation history numbers for the mono-energetic beam and the SOBP beam were set to 750 million particles and 2 billion particles, respectively.

The physical depth dose distribution data for the 155 MeV mono-energetic beam and the SOBP beam at PMRC measured using an electron diode field detector (EFD 3G-pSi, IBA Dosimetry) were used for comparison.

### Validation of the RBE-weighted dose by PHITS with implemented MKM

The calculation procedure for the RBE based on MKM coupled with PHITS was described in detail in reference [17]; thus, only an outline of the procedure is given here.

The RBE for surviving fraction *S* in a complex radiation field can be obtained by the equation:

(1) $$RBE_{C}\left(S\right)\;=\frac{D_{X}\left(S\right)}{D_{C}\left(S\right)}$$

where *D*_X_(*S*) and *D*_C_(*S*) are the doses used to obtain surviving fraction *S* in the X-ray and complex radiation fields, respectively. According to the linear quadratic (LQ) model, the dose used to obtain surviving fraction *S*, *D*(*S*), can be determined by the equation:

(2) $$D\left(S\right)\;=\frac{-\alpha +\sqrt{\alpha^{2}-4\beta \;\ln\left(S\right)}}{2\beta }$$

where α and β are the linear and quadratic coefficients of the LQ model, respectively. In the MKM, β can be regarded as a parameter independent of radiation quality, while α can be estimated from:

(3) $$\alpha \;=\;\alpha_{0}+\beta z_{1D}^{⁎}$$

where α_0_ is a constant that represents the slope of the surviving fraction curve in the limit of LET = 0. The parameter $z_{1D}^{⁎}$ denotes the saturation-corrected dose-mean specific energy, which can be calculated by:

(4) $$z_{1D}^{⁎}=\frac{l}{m}y^{⁎}=\frac{1}{{{\rho \pi r}_{d}}^{2}}y_{0}^{2}\int_{0}^{\infty }\frac{1-\exp\left(-y^{2}/y_{0}^{2}\right)}{y}d\left(y\right)\;dy$$

where ρ and r_d_ are the density and radius of domain, respectively, *y*_0_ is the saturation parameter, and *d*(*y*) is the dose distribution of *y* calculated by PHITS.

**Fig. 6.**
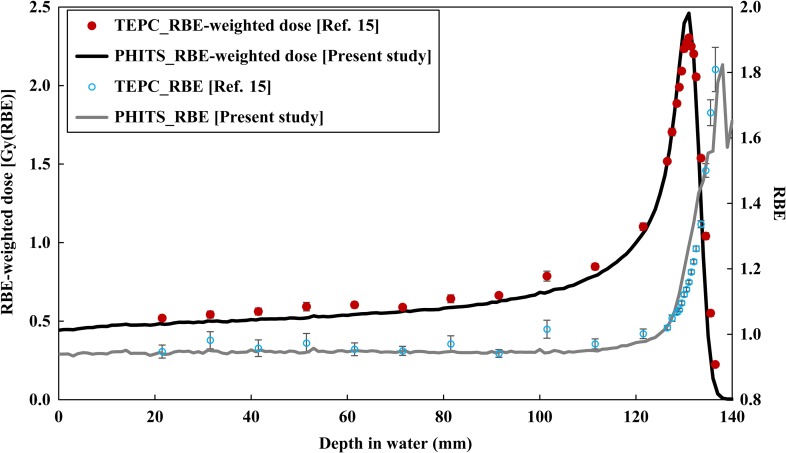
Comparison of RBE and RBE-weighted dose at the central axis in the water phantom after irradiation by the 155 MeV mono-energetic proton beam. The circles and lines represent the published data measured by TEPC [15] and calculated by PHITS, respectively. Error bars were taken from Ref. [15].

**Fig. 7.**
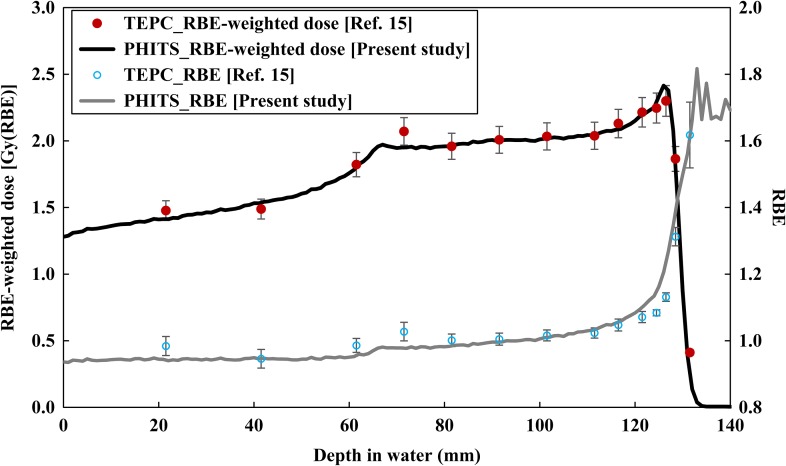
Comparison of RBE and RBE-weighted dose at the central axis in the water phantom after irradiation by the 155 MeV proton beam with 60 mm SOBP width. The circles and lines represent the published data measured by TEPC [15] and calculated by PHITS, respectively. Error bars were taken from Ref. [15].

In this study, RBE at each depth was evaluated by substituting 0.1 for S in Eq. (1), i.e. the RBE for the 10% surviving fraction was employed in the RBE-weighted dose estimation irrespective of the dose and dose rate. It is known that RBE depends not only on radiation quality but also largely on dose and dose rate. However, we ignored the latter factors in the same manner as done in the RBE-estimation method adopted for the HIMAC passive beam [40] as well as clinical proton therapy. The dose distribution of *y*, *yd*(*y*), for the mono-energetic and SOBP beams was calculated using the microdosimetric function of PHITS at 1 mm depth intervals. On the other hand, the active volume size of TEPC (diameter: 12.7 mm) is larger than the spatial resolution of PHITS. Therefore, the *yd(y)* spectrum calculated by PHITS at each depth was averaged over a range of ±1.5 mm centered at each interest depth of the beam axis. The diameter of 0.564 μm was selected to define the microdosimetric sensitive region to calculate *yd(y)* and the RBE-weighted dose based on the MKM. In estimating the RBE-weighted doses, the RBE for the 10% surviving fractions was determined from the calculated *yd*(*y*) for each depth, using the MKM parameters evaluated from the experimental surviving fractions of human salivary gland (HSG) cells irradiated with C, Ne, Si and Fe ions [16]. We employed 200 kVp X-rays (α value: 0.19 Gy^−1^, β value: 0.05 Gy^−2^ in the linear quadratic model) as reference radiation for calculating RBE [15, 16]. The values of α_0_, β, domain radius (r_d_) and saturation parameter (*y*_0_) for the MKM parameters used in this study were set as 0.155 Gy^−1^, 0.0615 Gy^−2^, 0.282 μm and 93.4 keV/μm, respectively, while those used to estimate the RBE-weighted doses were set as α_0_: 0.13 Gy^−1^, β: 0.05 Gy^−2^, r_d_: 0.42 μm, and *y*_0_: 150 keV/μm, as depicted in Figs 6 and 7. Note that we employed the same MKM parameters as used for carbon ion therapy [32], i.e. the parameters were not optimized for proton therapy.

## RESULTS

### Physical dose validation

**Fig. 2.**
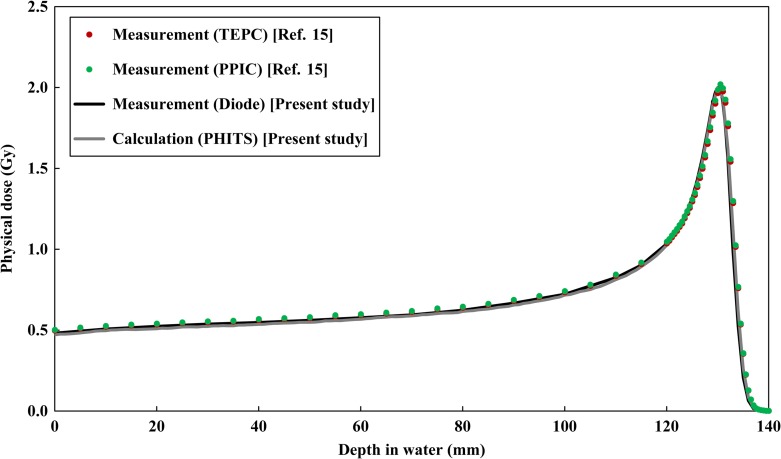
Comparison of depth physical dose distribution in the water phantom after irradiation by the 155 MeV mono-energetic proton beam. The circles and lines represent the published data and present data, respectively. The vertical axis shows the physical dose normalized at the Bragg peak as 2 Gy.

Figure 2 shows the physical depth dose distribution in a water phantom for the 155 MeV mono-energetic beam. In Fig. 2, the present study data (PHITS calculation and diode detector) are compared with the published data (TEPC, ionization chamber) [15]. The statistical uncertainties for the calculated values up to the distal fall-off of the Bragg peak were below 0.5%. Both the calculated and measured dose distributions in the present study were normalized at the Bragg-peak as 2 Gy. A maximum difference of 2.4% between the PHITS calculation and measurement with the electron diode detector was observed at the entrance of the water phantom.

**Fig. 3.**
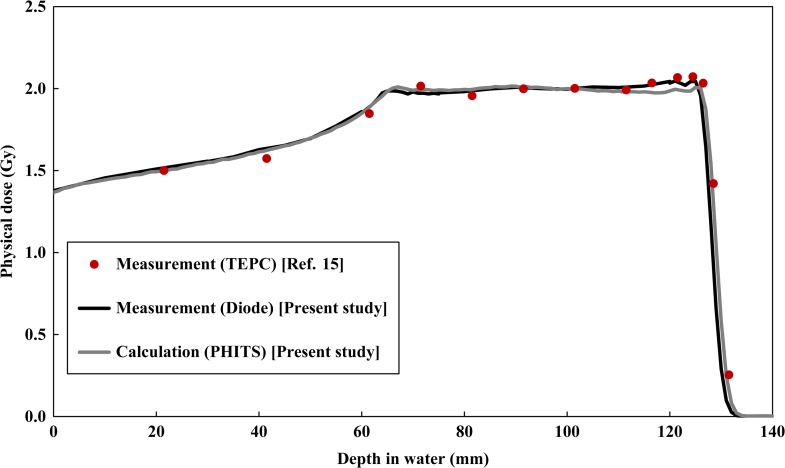
Comparison of depth physical dose distribution in the water phantom after irradiation by the 155 MeV proton beam with 60 mm SOBP width. The circles and lines represent the published data and present data, respectively. The vertical axis shows the physical dose normalized at the center of the SOBP as 2 Gy.

Figure 3 compares the physical depth dose distribution of the present study data (PHITS calculation and diode detector) with the published data for the 155 MeV SOBP beam [15]. Both the calculated and measured dose distributions in the present study were normalized at the center of the SOBP as 2 Gy. The statistical uncertainties for the calculated values up to the distal fall-off of the SOBP were below 0.5%. A maximum difference of 3.1% between the PHITS calculation and the measured data was observed at the distal of the SOBP.

### RBE-weighted dose validation

**Fig. 4.**
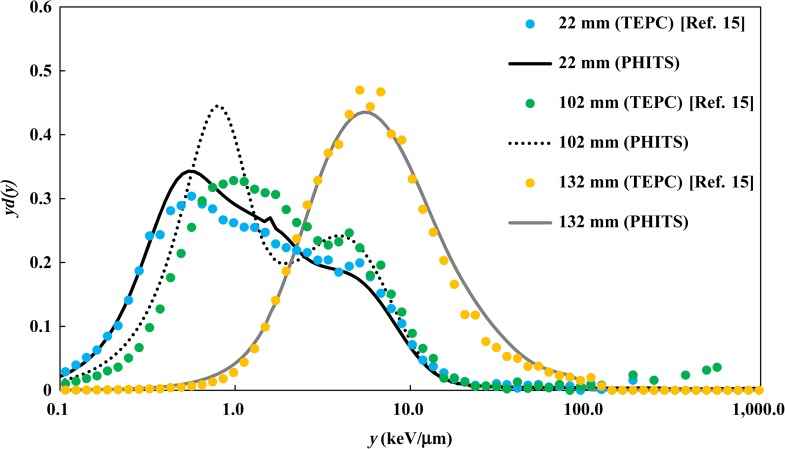
Comparison of *yd(y)* distributions in the water phantom after irradiation by the 155 MeV mono-energetic proton beam. The circles and lines represent the published data measured by TEPC [15] and calculated by PHITS, respectively.

**Fig. 5.**
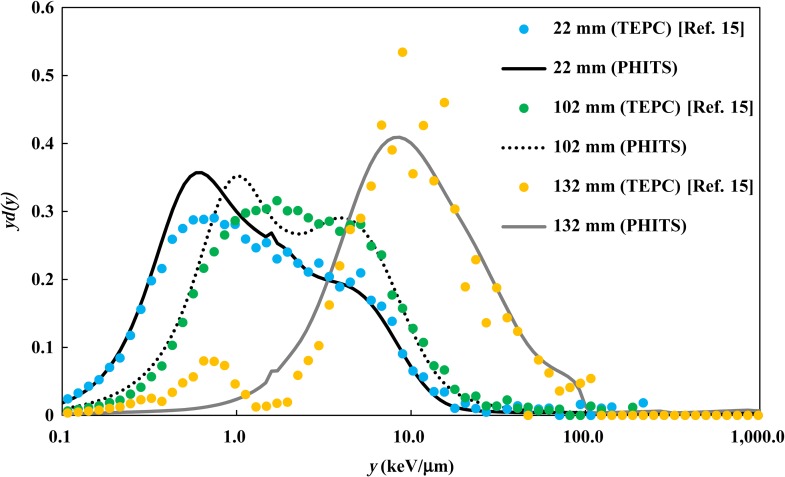
Comparison of *yd(y)* distributions in the water phantom after irradiation by the 155 MeV proton beam with 60 mm SOBP width. The circles and lines represent the published data measured by TEPC [15] and calculated by PHITS, respectively.

The *yd(y)* distributions calculated by PHITS were compared with the measured *yd(y)* at typical depths obtained by TEPC measurement. Note that the microdosimetric site diameter differed between the published experimental value and the PHITS calculation. Due to the difference in the site diameter, the calculated *y* distribution slightly shifted; however, it did not have much impact. Figures 4 and 5 compare the *yd(y)* distributions at three depths (22 mm, 102 mm and 132 mm) in the water phantom in the case of irradiation by the 155 MeV mono-energetic and SOBP beams, respectively. These depths correspond to the plateau region, the proximal of the Bragg peak region, and the Bragg peak region, respectively, for the mono-energetic beam, and the proximal, the center and the distal of the SOBP, respectively, for the SOBP beam. The *yd(y)* distribution shifts to higher *y* values at deeper regions because slower protons deposit more of their energy locally.

The calculated *yd(y)* distributions were generally in good agreement with the measurement data, except in higher *y* regions observed at the Bragg peak of the mono-energetic beam, where the calculation slightly overestimated the measured data.

Figures 6 and 7 compare the depth distributions of the RBE-weighted dose for the 155 MeV mono-energetic and SOBP beams, respectively. For the mono-energetic beam, the calculated dose distributions were normalized with the same factors used for normalizing the physical doses as shown in Fig. 2. With regard to the SOBP beam, the calculated RBE-weighted dose at the center of the SOBP was normalized to 2 Gy in order to compare it with the published data. The statistical uncertainties for all calculation data were below 0.8% up to the distal fall-off of the depth dose distribution. There was excellent agreement between the calculated and measured data, except in the Bragg peak region of the mono-energetic beam, where the calculation overestimated the measured data by 14.7%. This overestimation can possibly be attributed to the difference between the spatial resolutions of the calculation and the measurement because the physical doses dramatically vary with depth at the Bragg peak and overestimation of the RBE value due to the disagreement between the *yd(y)* distributions at higher *y* regions. Both the calculations and measurements suggested that the RBE-weighted doses in the SOBP region gradually increase with an increase in depth.

## DISCUSSION

In this research, we calculated physical and RBE-weighted dose distributions in a proton therapy facility with a fully mocked-up Monte Carlo calculation geometry. The physical dose distributions, *yd(y)* distributions, and RBE-weighted dose of the 155 MeV mono-energetic and SOBP beams calculated by PHITS agreed well with the experimental data, indicating that PHITS accurately simulates the proton beam line used for clinical proton therapy.

### RBE-weighted dose at the entrance of the water phantom for a mono-energetic beam

It is evident from Fig. 6 that the RBE-weighted dose at the entrance of a water phantom irradiated by a mono-energetic proton beam was slightly lower than 100% (93.9%), indicating that the RBE at the location was smaller than 1.0. This is because the ionization densities around the trajectory of the high-energy protons are generally smaller than those around the reference radiation adopted in this study, i.e. 200 kVp X-rays. Note that the RBE would be closer to 1.0 when higher energy photons such as γ-rays from ^60^Co are employed as the reference radiation, because the surviving fractions for photon irradiations generally become smaller with decreasing photon energy owing to the increase in dose-mean lineal energy [23]. This difference can be properly expressed by the MKM.

### Depth distribution of the survival fraction for the clinical SOBP beam

**Fig. 8.**
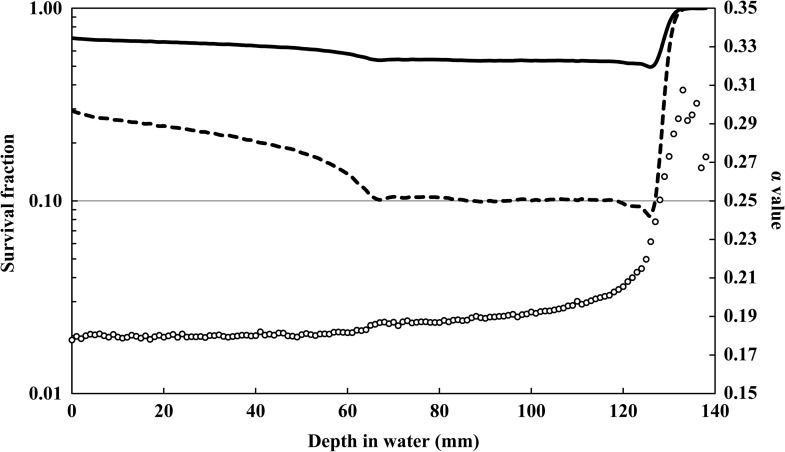
Calculated survival fraction of HSG cells along the central axis in the water phantom after irradiation by the 155 MeV SOBP beam. Solid line: Prescribed dose normalized as 2 Gy at center of SOBP. Dashed line: Survival fraction normalized as 10% at center of SOBP. Open symbols represent the calculated α values from the *y* distributions (right vertical axis).

We also estimated the corresponding depth distribution of the survival fraction of the HSG cells for the clinical SOBP beam from the *y* distributions calculated using the microdosimetric function of PHITS. For that purpose, the α value and $z_{1D}^{⁎}$ at each depth were calculated from the *y* distributions using Eqs (3) and (4), respectively. Figure 8 shows the depth distributions of the survival fraction for the clinical SOBP beam. The absolute values of the absorbed doses were normalized to 2 Gy at the SOBP center, or to yield 10% cell survival, i.e. 4.77 Gy, at the target in the center of SOBP.

The calculated α values at each depth obtained by this study were similar to the values reported by Kase *et al.* [15].

### Advantage of a *y*-based RBE estimator function

It is known that RBE varies with biological factors such as cell type and end point, and with physical factors such as dose level and beam quality [28, 41]. Several studies focusing on the relationship between RBE and beam quality in proton therapy have been reported [15, 42–44]. These studies have employed experimental or computational approaches as beam quality evaluation methods.

In order to evaluate the quality of the proton beams, linear energy transfer (LET) is more frequently used as the indicator of RBE than *y*. This is predominantly because LET is a non-stochastic quantity and its numerical value is easily calculable, while *y* is a stochastic quantity and its probability density is difficult to evaluate. However, the concept of LET cannot express differences in the track structure around the trajectory of charged particles due to δ-ray production. Thus, several experiments suggested that ion species dependences can be observed in the relationships between LET and RBE in surviving cell fractions (e.g. [45, 46]); RBE for lighter ions is generally higher than that for heavier ions with the same LET. Therefore, the RBE-estimator function for proton therapy should be adjusted when a LET-based model developed for carbon ion therapy is employed in the calculation. In contrast, the RBE-estimator functions based on *y*, such as the combination of PHITS and MKM, should be independent of the type of radiotherapy, because *y* can properly represent different track structures according to ion species. This feature is a great advantage of the *y*-based function, and is clearly demonstrated by the agreement between the calculated and measured RBE-weighted doses shown in Figs 6 and 7. It should be noted that the tendency of RBE to increase at the distal fall-off of the clinical SOBP beam has also been observed in different RBE-estimator functions based on LET [44, 47].

### Significance of this research and adaptation for future clinical application

This is the first study to validate the RBE-weighted dose for proton therapy by a combination of PHITS and MKM. This validation completes the computational microdosimetric approach based on the combination of PHITS and MKM for all types of clinical radiotherapy that require RBE evaluation, i.e. proton therapy, carbon ion therapy and BNCT.

The RBE-weighted dose calculation algorithm based on MKM was officially implemented in PHITS after version 2.89, allowing users to calculate the physical and RBE-weighted doses using default settings. This feature is a great advantage in adapting the calculations to TPS clinical applications.

A further direction of this study will be to establish the TPS that takes into account not only the depth distribution shown in this study but also the lateral distributions with various beam conditions using the RBE-estimator function in PHITS combined with clinical data for human body. It can be expected that this work will lead to an expansion of the adaptation of proton therapies like TOPAS with an RBE-weighted dose estimator based on Geant4 [8]. Moreover, a combination of PHITS and MKM is also expected to extend the usefulness of the RBE-estimator function in the TPS for all kinds of radiotherapies that require RBE-weighted dose estimation.

## CONFLICT OF INTEREST

The authors report no conflicts of interest.

## FUNDING

This work was supported in part by the Japan Society for the Promotion of Science (JSPS) KAKENHI Grant Number JP16K15343.
